# Radiopharmaceutical good practices: Regulation between hospital and industry

**DOI:** 10.3389/fnume.2022.990330

**Published:** 2022-09-07

**Authors:** Alain Faivre-Chauvet, Cécile Bourdeau, Mickaël Bourgeois

**Affiliations:** ^1^ Nantes-Angers Cancer Research Center CRCI2NA, University of Nantes, INSERM UMR1307, CNRS-ERL6075, Nantes, France; ^2^ Nuclear Medicine Department, University Hospital, Nantes, France; ^3^ Radiopharmacy Unit, ARRONAX Cyclotron, Saint-Herblain, France

**Keywords:** radiopharmaceutical, nuclear medicine, regulatory, good practices, hospital radiopharmacy unit, in-house

## Abstract

Radiopharmaceutical practices are divided into large-scale industrial manufacturing and small-scale “in-house” hospital radiopharmacy unit. The recent evolution of nuclear medicine involves deep consequences in this ever-present regulatory state, and hospital radiopharmacy units cannot be considered as contract manufacturing organizations (CMO). This review provides an updated status report of the official (and non-official) guidelines supporting the regulations required to meet hospital and industry common radiopharmaceutical manufacturing standards to facilitate the current and future innovative radiopharmaceutical development.

## Introduction

**Figure 1:**
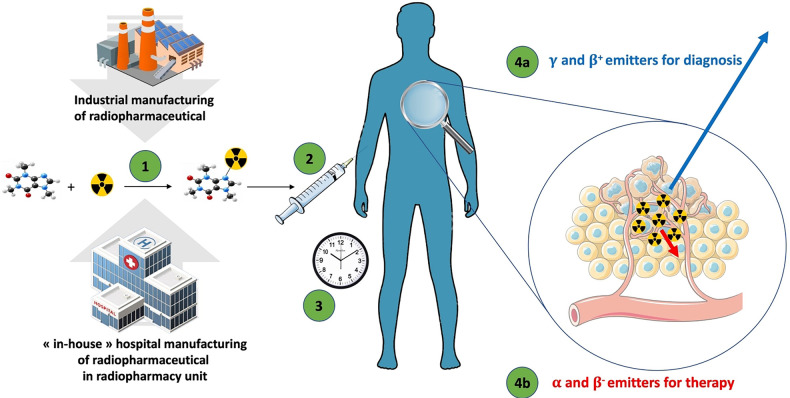
The general flow of radiopharmaceuticals. (1) The radiopharmaceutical compound is manufactured by the industry or “in-house” hospital radiopharmacy unit under GMP or PIC/S regulation, respectively. (2) The radiopharmaceutical compound is injected into the patient in the nuclear medicine department. (3) After the elapsed time needed for the specific pharmacological distribution of the radiopharmaceutical, the radioactivity is used depending on the purpose: (4a) an emission of radioactivity outside the body for external detection (diagnostic) with γ or β^+^ emitters; or (4b) local irradiation for therapeutic purpose with α, β^−^, or Auger emitters.

The original idea of using radioactive compounds for medical purposes emerged from a close collaboration between physicists and physicians in the mid-1930s in Boston, and the first-in-human (FIH) utilization of radioactive iodine-131 ([^131^I]) was done in 1941. Iodine-131 was first used for the diagnosis and care of patients with thyroid dysfunction (1); consecutively, nuclear medicine emerged as a medical specialty. With growing knowledge of pharmacology and radiochemistry, new organic compounds and radiolabeled biologically active pharmaceutical ingredients (API) named “radiopharmaceuticals” have been designed to expand the field of nuclear medicine to organs other than the thyroid gland for both diagnostic and therapeutic purposes (Figure 1).

Since the introduction of nuclear medicine, the variety of available medical radionuclides was limited in terms of nuclear production, supply, and logistics capability for the first three decades. Initially, the great majority of radioisotopes were produced in nuclear research reactors for industrial users, and radiopharmaceutical manufacturing was performed by industries under stringent manufacturing and quality control (later called Good Manufacturing Practices rules - GMP) with marketing authorization. Despite this classical approach to pharmaceutical supply, an exception appeared in the mid-1960s with the availability of Technetium-99m ([^99m^Tc]) in radiopharmaceutical practice. Technetium-99m has many benefits for nuclear medicine applications: physical half-life compatible with biological processes, photon energy readily detectable by gamma cameras, low radiation for patients, and great variability of pharmaceutical compounds able to complex [^99m^Tc]. However, the short half-life of [^99m^Tc] (6 h) is a logistical constraint for a low-cost supply chain worldwide. To circumvent this inconvenience, industrial suppliers of medical radioisotopes used molybdenum-99 ([^99^Mo]) with a longer half-life, which decays to [^99m^Tc]. Molybdenum-99 is placed in a generator system by the industry, and the ^99m^Tc isotope can then be recovered in a local radiopharmaceutical hospital for “in-house” preparation of various radiopharmaceutical compounds. The availability of [^99^Mo]/[^99m^Tc] generators with pharmaceutical quality was the starting point of nuclear medicine worldwide in the 1970s (2).

Since the first radiopharmacy historical period and despite the worldwide disparities between each national governance, radiopharmaceutical manufacturing can be currently divided into two different regulatory frameworks: “the GMP regulation for commercial suppliers” (radiopharmaceuticals with marketing authorization) and “Guide to good practices for the preparation of medicinal products in healthcare establishment” promulgated by the Pharmaceutical Inspection Co-operation Scheme (PIC/S) (3) for the “in-house” radiopharmaceutical preparation by hospital radiopharmacy units. This historical division of industrial production and hospital preparation of radiopharmaceutical is still relevant today and is at the origin of regulatory and technical constraints when setting up industrial promoting clinical trial with an hospital preparation of the investigated radiopharmaceutical. The main objective of this article is to provide possible solutions to bridge the remaining gap between hospital and industry to facilitate the communication between each partners around the production (and quality control) prerequisites.

### Recent evolution and problems

The current routine practice in nuclear medicine is still mainly driven by “in-house” radiolabeling kits, where radionuclides are manually added by the hospital radiopharmaceutical units in sterile conditions under radio safety precautions to prepare the final radiopharmaceutical compounds closest to the patient use. The evolution of nuclear medicine (and associated radiopharmaceuticals) in neurology and oncology with new and innovative isotopes has necessitated the deployment of radionuclide production facilities. To meet this demand, particle accelerators were built around the world to produce interesting radioisotopes, and a cyclotron network was built close to nuclear medicine departments. This expansion of production sites was carried out in the historical continuity of radiopharmacy, and the cyclotrons were placed, according to their locations, under the responsibility of industrial and/or healthcare establishments.

Concerning industrial manufacturing, radiopharmaceutical production must be comply with both pharmaceutical and radiation protection regulations. The place and impact of specific national nuclear regulations are beyond the scope of this article. For the pharmaceutical aspects, the ICHQ7 “good manufacturing practice (GMP) for active pharmaceutical ingredients (API)”, edited by the International Council for Harmonization of technical requirements for pharmaceuticals (ICH), gathers all the prerequisites to perform a radioactive API used in the composition of radiopharmaceutical drug by industrial production. Consequently, the commercial supplier must follow all the official GMP guidelines that endorse specific guidelines for the manufacture of radiopharmaceutical products [e.g., Annex 3 for the European Union (4) or 21 CFR parts 211 and 212 for the United States (5)].

However, the current trend for innovative radiopharmaceutical development mainly involves manufacturing in hospital radiopharmaceutical units, and all recent breakthroughs in clinical nuclear medicine are based on “in-house” manufacturing. This is a direct consequence of the radiopharmaceutical particularities (Table 1), which often require a manufacturer to be as close to the patient as possible. Thus, many radiopharmaceutical clinical trials involve healthcare establishment and industrial partnership with a particular regulatory stumbling block around the preparation, control, and release of radiopharmaceuticals. Indeed, the “in-house” manufacturing of radiopharmaceuticals by hospital radiopharmaceutical units can be defined by the official PIC/S, described earlier. The objective of the PIC/S is to be officially associated with scientific and technical goals, where the members include more than 50 health agencies worldwide but without binding arrangements. Consequently, it is difficult or even impossible for a hospital radiopharmaceutical unit to obtain a GMP certificate (or equivalent) from its national health agency, and there is no official recognition of the PIC/S regulation framework by pharmaceutical industries.

**Table 1:** Radiopharmaceutical particularities.

Specificities of radiopharmaceutical products	Consequences
Radioactivity	• Radiation protection rules • Specific knowledge and highly specialized staff • Expensive to develop a dedicated line for one product (need of line clearance between two productions)
Short radioactive half-life and short shelf-life product	• Fast logistic • No long-term storage • Extemporaneous manufacturing • Not fully tested before release (especially for sterility tests which are obtained after injection of the product)
Small-scale production (for hospital production)	• Good adaptation to targeted diagnosis/therapy • Financial difficulties for clinical trials • Low volume (limitations to perform extensive analytical evaluation)
Tracer amount	• Low quantity (leakage of pharmacological effect, low toxicity risk with micro-dosing concept)
Unlicensed starting materials (chemical precursors)	• Implementation of the IMPD with raw material quality control

For clinical trials that require the manufacture of radiopharmaceuticals, this regulatory discordance is particularly prominent around investigational medicinal product dossier (IMPD) module 3 requirements (6–8). This part of the IMPD concerns all the detailed chemistry, manufacturing, and control (CMC) aspects of the innovative radiopharmaceutical. Due to the specific regulation of “in-house” manufacturing, the hospital radiopharmaceutical unit could not be considered a classical contract manufacturing organization (CMO) or contract development and manufacturing organization (CDMO) by the industry, making it difficult to set up clinical trials. These difficulties are mainly driven by an administrative blockage (hospitals don't have industrial status and, from an administrative point of view, their respective health agencies can't give this status to healtchcare establishment). The classical current way to solve this problem consist in a common writing of a Quality Technical Agreement (QTA) where each partners stipulate their expectations. At this step it is up to the pharmaceutical industry to accept (or not accept) the risk of a unrecognized GMP-labeled production in an healtcare establishment. This QTA step remains frequently a difficult point in term of misunderstanding expectation with both regulatory and quality department of industrial promoter which are not familiar with the radiopharmaceutical specificities.

### Evolution in close future and harmonization perspectives

Because of the evolution of the radiopharmaceutical needs and to provide an appropriate response to both the radiological protection regulation and the robustness needed for clinical trial production, we have been observing for a few years a standardization of the practices with the availability on the market of GMP kits for the extemporaneous “in-house” radiolabeling of radiopharmaceuticals. In parallel, we currently observe a generalization of the use of automated synthesis module in hospital radiopharmaceutical units. Automated modules are devices that can perform a sequence of radiochemical manipulations automatically. It consists of an interconnected tubing network of vials that can automatically transfer a solution from different starting vials and mix the solution under different temperatures, and the desired final product is sterilized by a final filtration (9). In addition to the evident radiation dosimetry gain brought about by non-manual manufacturing, automated radiosynthesis offers various advantages for radiopharmacy units, such as fast-line clearance between two preparations, good reliability, repeatability, and traceability in manufacturing. This generalized use of automatized technology appears to be a key factor and paves the way for the harmonization of industrial and “in-house” manufacturing practices.

To bridge the remaining regulatory gap (10), some major nuclear medicine knowledgeable societies in the US and the European Union have started, from several years already, to publish some indications (11–14) to facilitate the approval by the authorities as well as recommendations for their members allowing to meet quality requirements on certain aspects, such as quality risk assessment (15), process qualification of small-scale production (16, 17) or validation of analytical methods (18). Although these guidelines remain non-official recommendations, they present the advantage to meet several industrial specifications and facilitate relationships between partners. Despite the great interest of these guidelines, they remain different and out-of-scope of the official GMP used as legal reference in the conventional pharmaceutic industries (i.e. non-radioactive).

Furthermore, the initial step towards the achievement of officials recognized that good practices for radiopharmaceuticals are currently in progress. These guidelines are currently being drafted by the World Health Organization (WHO) in partnership with the International Atomic Energy Agency (IAEA) (19). The main objective of this draft is to provide dedicated guidance for the manufacture of investigational radiopharmaceuticals used in both early and late clinical trials to fulfill the challenges of the rapidly expanding field of molecular imaging and targeted radiopharmaceutical therapy.

## Discussion

During the last decade, the need for personalized and precision medicine has grown, and theranostic approaches in nuclear medicine appear to be promising solutions for tailored patient treatment, particularly in oncology. This rapid evolution has a direct impact on radiopharmaceutical practices, which industrial and commercial large-scale manufacturing cannot adequately address because of the specificity of radiopharmaceuticals. To support this renewed interest, industrial companies turned their attention to the “in-house” small-scale preparation capacities of the radiopharmacy units of healthcare establishments. Unfortunately, the current hospital regulatory frame is dependent on national governance disparities, and this lack of harmonization and recognition is a hurdle for clinical trial settings.

To limit this regulatory and technical bottleneck in the passage of radiopharmaceutical compounds to their clinical applications, it is mandatory to continue and enforce the development of strong tripartite collaboration between the industrial, hospital, and regulatory partners to provide a common and recognized framework of guidelines based on specific risk assessment, bearing in mind the need for flexibility and responsiveness in the development of innovative radiopharmaceuticals. The position of hospital radiopharmacy and the “in-house” preparation of radiopharmaceuticals is an important and very specific point and deserves a debate with the worldwide regulators to homogeneate the guidelines and thus facilitate the hospital/industry interface. The key to future radiopharmacy success strongly depends on successful harmonization to provide fast development, financial attractiveness, safety, and effective radiopharmaceuticals by “in-house” hospital radiopharmacy units for industrial or institutional multicentric clinical trials. This success, for the benefit of our patients, can only be achieved with the involvement of the different contributors involved at every level in the radiopharmaceutical clinical trials from manufacturing to regulatory approval.
